# Post-lockdown changes in diet in Italy and the USA: Return to old habits or structural changes?

**DOI:** 10.1186/s40100-022-00234-7

**Published:** 2022-10-14

**Authors:** Gabriele Scozzafava, Caterina Contini, Francesca Gerini, Leonardo Casini

**Affiliations:** Department of Agriculture, Food, Environment and Forestry - DAGRI, University of Firenze, Piazzale delle Cascine 18, 50144 Florence, Italy

## Abstract

This study analyses the impacts of the COVID-19 pandemic on food consumption at the end of the first lockdown in the New York State (USA) and in Italy (spring 2020). The results of our study show that important changes occurred in food habits in these two countries, in which lockdown was very similar. Three models of response to the shock of the lockdown were noted in both countries. The first model (40%) includes individuals who largely increased their food consumption, the second model (26%) showed a more virtuous and responsible behaviour, while the third model (34%) displayed no change in food consumption. Diet quality in terms of healthiness and sustainability declined in the USA, while in Italy, approximately one-third of the sample showed an improvement in diet in these same areas. The use of sociodemographic, motivational, and behavioural variables to profile subjects who adhered to each food model has made it possible to obtain information that can be used to develop communication campaigns and policies for a healthier and more sustainable diet.

## Introduction

In an attempt to slow the spread of COVID-19, countries such as China and Italy implemented lockdowns and issued a series of restrictions intended to limit social contact among individuals. These measures involved the total shutdown of schools, universities, offices, museums, movie theatres, theatres, bars, restaurants, gyms, business activities, and shops operating in the production and/or sale of nonessential commodities. During the lockdown, people’s freedom of movement was limited, and they were permitted to leave their homes only to work, to attend to health matters, and to obtain necessities such as groceries. In Italy, the general lockdown in response to the first wave of the coronavirus was implemented via the #iorestoacasa (I stay home) decree dated 9 March 2020 and took effect on 10 March 2020. Business and hospitality industry activities resumed gradually on 18 May 2020. Other countries, such as the USA, adopted less extensive strategies, applying a lockdown to more limited areas and contexts. For example, New York State underwent a veritable lockdown that lasted from March to June 2020.

In addition to harmful effects on public health and the economy, the pandemic also had inevitable repercussions on the social level (Donthu and Gustafsson 2020; Zambrano-Monserrate et al. 2020), with the lockdown constituting a shock that upset the habits of millions of people, modifying their behaviour and making them reconsider their priorities. In particular, the shutdown of businesses and schools, the modality of “working from home”, and greater time spent in the home brought about transformations in people’s lifestyles and eating habits (e.g. Cranfield 2020; Hobbs 2020; Rodríguez-Pérez et al. 2020; Fanelli 2021; Izzo et al. 2021; Marty et al. 2021).

The literature from numerous studies has analysed how exceptional and shocking events can bring about changes in people’s diets. Wars, financial crises, and natural disasters have historically set off dynamics in both the food supply chains and the psychological and economic conditions of consumers that lead to changes in dietary choices (Henderson et al. 2010). For instance, during World War II, food became a luxury item because of its limited availability to families (Flessig 2020). A study conducted in Great Britain found that during the conflict, the diet was based primarily on the consumption of carbohydrates such as potatoes and grains, while proteins and fats of animal origin formed only a limited portion (Hollingsworth 1961). The same author notes that it took several years for people’s diets to improve in terms of both quality and quantity of foods consumed. These trends can be attributed to the ineffectiveness of supply chains, the rationing of foods regulated by the British government, and the economic conditions of families. Ten years after the end of the war, there was an improvement in the nutritional value of the diet with increased consumption of fruit, vegetables, meat, butter, and milk. Furthermore, an increased energy content in meals was observed compared with the period before the conflict.

Dietary habits have also worsened after recessions (Foscolou et al. 2017; Bonaccio et al. 2018). In many European countries, the economic crisis of 2008 was followed by a period in which many consumers could not afford to purchase meat, chicken, or fish (Davis and Geiger 2017). A study conducted in Italy found that consumers who perceived the effects of the recession negatively reduced their observance of the Mediterranean diet and purchased foods of lesser quality. Lower socio-economic groups, in particular, recorded more negative changes in diet quality (Bonaccio et al. 2012). While changes in dietary habits in these circumstances seem to be mostly caused by economic difficulties, in the cases of natural disasters, such as earthquakes, tsunamis, and hurricanes, changes in dietary behaviour are also attributable to emotional states. These episodes are, in fact, followed by phenomena of overeating and emotional eating, which have led to increased consumption of so-called comfort foods rich in sugars and fats which are capable of inducing pleasure (Adam and Epel 2007; Henderson et al. 2010; Kuijer and Boyce 2012; Ma et al. 2021). The principal factors that have influenced dietary habits were living in evacuation shelters, lack of access to stores, and social isolation (Goryoda et al. 2018).

In the case of COVID-19, changes in the dietary habits of families during the lockdown occurred in almost all countries taking part in shared general patterns, as well as in disparate and, at times, contrasting behaviours of consumers with respect to the healthiness and environmental sustainability of the diet (e.g. Di Renzo et al. 2020; Marty et al. 2021; Murphy et al. 2021). No longer able to eat in cafeterias, restaurants or bars during the lockdown period, people prepared and consumed their meals at home. This brought about a considerable and generalized increase in supermarket purchases (Bracale and Vaccaro 2020; Leone et al. 2020). Even the way people shopped changed during the period of restrictions (Martin-Neuninger and Ruby 2020). Many consumers who were unable to go to their usual shops had to go to those closer to home, even if these shops were more expensive and offered less variety of products. Furthermore, to avoid the long lines at the entrance of supermarkets caused by the restricted number of in-store shoppers, consumers preferred to go to stores less often and chose instead to stock up when they did go. Other trends that gained ground during quarantine include avoidance of going to the supermarket and the purchase of products at lower prices, the strong increase in online purchases of groceries, and the reliance on home delivery of ready-to-eat foods (ISMEA 2020; Osservatorio Business B2C 2020; Redman 2020). From the viewpoint of food choices, the inability to shop every day caused a reduction in the consumption of fresh foods such as fruit, vegetables, and fish (Fanelli 2021; Mitchell et al. 2021) and an increase in purchases of products with longer shelf lives such as canned products, dry legumes, ultrahigh temperature processed milk, frozen foods, and pasta (Bracale and Vaccaro 2020; Tan et al. 2020). During the lockdown, increased consumption of red meat was observed, both packaged and tinned, and of cold cuts in many countries such as Italy (Bracale and Vaccaro 2020), France (Marty et al. 2021), and the USA (Mitchell et al. 2021). Regarding the consumption of convenience foods, similar trends were observed in many countries, including Italy, France, Great Britain, the USA, Ireland, and New Zealand (Fanelli 2021; Marty et al. 2021; Murphy et al. 2021), where this type of food product showed an increase in consumption. With more time on their hands and the need to escape boredom, people were driven to spend more time cooking, even preparing more laborious dishes such as pizza, fresh pasta, bread, and sweets (Di Renzo et al. 2020; Marty et al. 2021). Before the lockdown, many consumers purchased ready-to-eat foods to save more time for work or hobbies, but during quarantine, this necessity disappeared. Another trend that emerged during the lockdown period was the diffusion of emotional states such as fear, anxiety, and depression, which influenced dietary choices and strengthened the phenomenon of emotional eating (Termorshuizen et al. 2020; Gómez-Corona et al. 2021; Molina-Montes et al. 2021). Recent international studies have indeed observed an increase in the consumption of sweets, salted snacks, and soft drinks during the lockdown (e.g. Bracale and Vaccaro 2020; Leone et al. 2020; Robinson et al. 2021). This eating pattern, defined by the consumption of foods with a high caloric content, exerts a negative effect on the quality and healthiness of foods. Precisely because of the changes in the makeup and quantity of foods consumed and in limited physical activity, a high percentage of consumers have stated that they have put on weight (e.g. Izzo et al. 2021; Marty et al. 2021; Robinson et al. 2021).

Changes in dietary choices during the lockdown thus seem to be characterized by a tendency toward unhealthy dietary behaviours, which leads to concerns around people’s health. On the other hand, this phenomenon is not true in absolute terms. Several consumer groups have, in fact, assumed virtuous dietary habits, be it in terms of diet healthiness through a greater consumption of fruits, vegetables, and legumes (CREA 2020; Ellison et al. 2021) or in terms of greater attention to environmental sustainability (Cavallo et al. 2020; Pappalardo et al. 2020; Švecová et al. 2020), along with a reduction in food waste (Roe et al. 2021). Regarding the consumption of alcoholic beverages, mixed trends were also observed during the lockdown. In the USA (Mitchell et al. 2021) and in the Netherlands (Poelman et al. 2021), consumers stated that they consumed the same quantity of alcoholic beverages as in previous periods. A decline in consumption was observed in France (Marty et al. 2021), while the opposite phenomenon was found to occur in Italy (Izzo et al. 2021).

While the literature that has studied dietary behaviours during the lockdown has stressed a heterogeneous picture, limited information is available about what occurred after the end of the restrictions. Except for the study by Zhang et al. (2020) that points out that irrational and unhealthy behaviours occurring during the lockdown in China have only partially improved, to our knowledge, no study has directly examined how dietary behaviours changed after the lockdowns. It is therefore important to delve deeper into these aspects and verify whether the changes caused by the shock of the lockdown have consolidated and led to more structural changes in various contexts.

Our study focuses on this context and has the objective of pointing out the impacts of the COVID-19 pandemic on food consumption in Italy and in the USA (limited to New York State) after the lockdowns that ended in the spring 2020. We chose to focus on these two countries since they are very different in terms of sociocultural and behavioural characteristics, as well as in terms of attention to food and diet. Studying the consequences of the lockdown on people’s diets in these two different countries appears particularly significant and useful in understanding how the same phenomenon can have differential impacts on food consumption.

This study specifically calls attention to the changes in food habits with respect to healthiness and sustainability, provides of a better understanding of the consumer’s dietary behaviour, and delineates strategic information for the development of communication campaigns and policies for a healthier and more sustainable diet.

These objectives are explained more clearly in the following hypotheses:

### H1

The lockdown has induced changes in dietary habits that have been maintained even after the end of restrictions both in Italy and in the USA.

### H2

The lockdown has not caused homogeneous changes in dietary behaviours related to health and sustainability.

To take into account the possible heterogeneity of the changes, we carried out a segmentation of the sample in order to identify different types of change and understand the drivers of each of these. The literature regarding dietary changes shows how the study of this phenomenon cannot preclude an analysis of the diet preceding the change (Marty et al. 2021; Caso et al. 2022; Gerini et al. 2022; Mignogna et al. 2022). This leads us to our third hypothesis:

### H3

The variations in dietary behaviours after lockdown depend on the diet of the pre-pandemic period.

In addition, in the literature dietary changes are traditionally interpreted in relation to sociodemographic characteristics (see the review by Aertsens et al. (2009)). We therefore hypothesize the following:

### H4

The variations in dietary behaviours after lockdown depend on sociodemographic characteristics.

Another approach for analysing dietary changes is based on the study of the motivations that underlie food choices and consumption. In addition to sociodemographic and psychographic variables, understanding food choice motives (FCM) provides valuable insights for explaining consumer behaviour (Verain et al. 2021). Food choice motives can be grouped into different categories such as health, mood, convenience, sensory appeal, natural content, price, weight control, familiarity and ethical concerns (Steptoe et al 1995). In this context, Onwezen et al. (2019) have developed a theoretical framework that evaluates motivations related to food choices using a single-item measurement. This approach consists of eleven measurements that include both individualistic and collective objectives. This methodology provides the practical advantage of being a shorter and reliable option for analysing the predictive value of food choice motivations. Using the framework proposed by Onwezen et al. (2019), we hypothesize the following:

### H5

The variations in dietary behaviours after lockdown depend on food choice motives.

The identification of groups of consumers whose diet has worsened or improved can enable the public decision-makers to understand the variables and the conditions that have promoted disparate dietary behaviours and to develop actions to sensitize specific targets within the population.

## Materials and methods

### The survey and recruitment

To test Hypotheses H1-H5, we analysed data obtained through an online survey conducted in September 2020. This month was representative of a post-lockdown situation. In fact, despite the shutdowns after this date due to the re-exacerbation of the health crisis, in September 2020, the pandemic was under control in the two countries of our survey, in terms both of the number of infected people and deaths and the number of those hospitalized. This aspect is very important because despite the successive appearance of other waves of the pandemic, at that time, the situation seemed almost to have returned to normal. The questionnaire was administered by an international marketing research company (Toluna, Wilton, Connecticut, USA, Inc.) to its online panel. The sample consisted of 1229 respondents, including 726 from Italy and 503 from the USA. As regards respondents from the USA, a filter was applied to consider only those residing in the State of New York, since that was the only area where the lockdown enforced was similar to Italy’s in terms of duration and type of restrictions (ABC 2020; Presidency of the Council of Ministers 2022).

The procedures performed in this study are in accordance with the ethical standards of the institutional and/or national research committee and with the 1975 Helsinki declaration and its later amendments or comparable ethical standards. Informed consent was obtained from all participants involved in the research.

The first part of the questionnaire focused on changes in food consumption compared with the period before the coming of COVID-19 and after the shock of the lockdown with respect to different categories of foods, beverages, methods of conservation, and sales points, which cover the food habits in Italy and USA. The changes in diet were recorded by asking respondents to refer to their diet pre- and post-lockdown and to indicate for this period the percentage variation in quantity consumed of 21 food categories (i.e. Grains, Vegetables and fruit, Legumes, Potatoes, Fish, Processed meat, Red meat, Pork meat, Sweets, Salted snacks, White meat, Eggs, Milk, Dairy products and cheeses, Wine, Beer, Soft drinks and fruit juices, Spirits, Ready to eat, Frozen food, and Canned food). The survey used a scroll bar with a variation range from − 100% to + 300%.

Subsequently, we recorded the quality of the diet before COVID-19 using the approach developed by Cavaliere et al. (2018), which refers to the Mediterranean diet as the model of a healthy, balanced, and sustainable diet. This procedure measures food quality by calculating adherence to the Mediterranean diet (MD) defined by the MD pyramid guidelines (Dernini and Berry 2015; IFMeD 2016; Dernini et al., 2017).

Specifically, respondents were asked to specify the frequency with which they consumed the following food categories in a typical week before COVID: Grains, Vegetables and fruit, Legumes, Potatoes, Fish, Processed meat, Red meat, Pork meat, Sweets, Salted snacks, White meat, Eggs, Milk, Dairy products and cheeses. The survey used a 5-point Likert scale (1 = Never; 2 = Less than once a week; 3 = A few times a week; 4 = Once a day; 5 = More than once a day). We calculated the MD Index by giving a score ranging from 0 to 2 to each food category (where 0 corresponds to low adherence and 2 to high adherence) (Cavaliere et al. 2018). By summing these scores in all the categories considered, we obtained an MD Index for each respondent. The criteria for assigning the adherence scores derive from the study by Cavaliere et al. (2018). The maximum achievable score is 28.

Finally, we completed the survey by collecting data on the frequency with which the respondents consumed the following food categories not included in the MD Index: Wine, Beer, Soft drinks, Spirits, Ready to eat, Frozen food, and Canned food. The collection method was the same as that used for the categories comprising the MD Index.

In the second section of the survey, we collected information about food choice motives using the scale of the single-item Food Choice Motives Questionnaire developed by Onwezen et al. (2019), which detects the importance of food purchasing motivations divided into eleven measurements. In particular, we asked respondents to indicate their degree of agreement with the following statements “It is important to me that the food I eat on a typical day …”: is healthy, is a way of monitoring my state of mind, is quick, provides me with pleasurable sensations, is natural, is affordable, helps me control my weight, is familiar, is environmentally friendly, is animal friendly, and is fairly traded. The survey used a 5-point Likert scale, where 1 corresponds to “Strongly disagree” and 5 to “Strongly agree”.

Finally, the third part of the questionnaire focused on sociodemographic questions.

### Segmentation and profiling

On the basis of the variations in quantities consumed before and after lockdown in the 21 categories studied, we have identified homogeneous segments of consumers with regard to dietary behaviours by employing a pooled model of the data from the two countries. This approach made it possible to underline the main overall adaptive responses in food habits that were brought on by the pandemic, regardless of sociodemographic, psychographic, and behavioural characteristics.

The procedure that was followed to define the segments began with an analysis of the principal components (PCA) carried out on the percentage variations in quantities consumed in the 21 categories studied in the post-lockdown period compared with before the pandemic. The PCA supplied the individual factor loadings upon which a cluster analysis was implemented and made it possible to characterize the diet of each individual, reducing the number of variables that describe them and limiting the loss of information as much as possible. The latent variables identified in this manner describe and interpret the dietary behaviour changes of each individual, considering only those foods that effectively succeed in explaining most of their variance.

The factor loadings associated with each component were then utilized to perform the actual identification of segments of consumers characterized by homogeneous consumption models. The procedure adopted is that of clustering. Cluster analysis consists of a set of statistical techniques capable of identifying groups of units similar to one another, according to a specific criterion with respect to a set of characteristics taken into consideration. The objective is to aggregate disparate units into subsets that tend to be homogenous and mutually exhaustive. In our case, the aggregation criterion utilized relates to Ward’s hierarchical method^1^ (Johnson 1967; Everitt 1979), which provides for the reunification, at every stage of the process for the two groups whose fusion produces the minimum increment possible of internal deviance.

We identified the optimum number of clusters by analysing the Caliński–Harabasz pseudo-F index and the Duda–Hart Je(2)/Je(1) index (Duda and Hart 1973; Caliński and Harabasz 1974). The Caliński–Harabasz index suggests a solution with two clusters, while the Duda–Hart index indicates a solution with three clusters. The latter choice provides more detailed information on dietary behaviours compared with the one with only two clusters and we therefore opted for the three-cluster solution.

Sociodemographic and psychographic variables were instead used to profile homogeneous behavioural classes. Specifically, by using the chi-squared automatic interaction detection (CHAID) methodology (Kass 1980), we were able to identify the variables capable of significantly characterizing each cluster identified. CHAID uses an algorithm based on several steps that utilize chi-squared statistics. Various partitions of each independent variable are tested with this algorithm, and the partition that identifies the difference between the classes in a more statistically meaningful manner is selected. For a more detailed description of the method, reference should be made to Kass (1980). CHAID has numerous advantages such as, for example, the possibility of using nominal and interval variables as predictors and of choosing continuous variables as criterion variables (Díaz-Pérez and Bethencourt-Cejas 2016).

We have thereby been able to identify the main models of response to the pandemic that are shared by both countries and, at the same time, to investigate the differences in the performance of each model in different contexts. This would have been far more difficult if we had acted on models estimated for each country, in which case the comparison would have been complicated by differences in the variations of the consumption of the different foods that form part of the identified food habits.

## Results

Table 1 shows the demographic characteristics of the sample we considered in our study compared to the population of Italy and USA. The sample is representative of the Italian and NY populations by gender.

**Table 1:** Demographic characteristics of our sample

	Italy		NY State		Total sample	
	Absolute Figures	%	Absolute Figures	%	Absolute Figures	%
*Gender*						
Male	351	48 (49)	229	46 (49)	580	47
Female	375	52 (51)	274	54 (51)	649	53
*Age*						
18–34	161	22 (23)	137	27 (31)	298	24
35–54	291	40 (36)	221	44 (33)	512	40
> 55	274	38 (41)	145	29 (36)	419	36
*Number of household members*						
1	68	9	101	20	169	14
2–3	412	57	248	49	660	54
4+	246	34	154	31	400	32
*Number of children under 18 years old in the household*						
0	68	9	100	20	168	14
1–2	558	77	277	55	835	68
3+	100	14	126	25	226	18
*Total respondents*	726	59	503	41	1229	100

Census data referring to Italy (ISTAT 2022) and State of New York (U.S. Census Bureau 2022) are shown in brackets

Data were first processed regarding the frequency of consumption before COVID-19, which confirmed the presence of different dietary habits in Italy and the USA, delineating two initially heterogeneous realities that suffered the same shock, that of the lockdown. Table 2 compares the average frequency of consumption of the sample analysed and in the two countries considered before the pandemic arrived.

**Table 2:** Pre-COVID-19 average food consumption frequencies in Italy and the USA

Food categories	Total sample		Italy		USA	
	Mean	SD	Mean	SD	Mean	SD
Grains	3.51	0.94	3.75	0.84	3.18	0.98
Vegetables and fruit	3.82	0.90	3.93	0.87	3.67	0.94
Legumes	2.70	0.89	2.92	0.66	2.38	1.07
Potatoes	2.87	0.71	2.88	0.60	2.86	0.84
Fish	2.69	0.81	2.76	0.65	2.59	1.00
Processed meat	2.75	0.86	2.90	0.74	2.52	0.98
Red meat	2.68	0.78	2.69	0.69	2.65	0.91
Pork meat	2.44	0.82	2.57	0.70	2.26	0.93
Sweets	3.03	0.90	3.03	0.85	3.03	0.96
Salted snacks	2.81	0.92	2.69	0.86	2.97	0.97
White meat	2.97	0.74	2.98	0.62	2.96	0.88
Eggs	2.96	0.78	2.86	0.62	3.11	0.93
Milk	3.41	1.11	3.46	1.05	3.33	1.19
Dairy products and cheeses	3.27	0.81	3.26	0.73	3.27	0.93
Wine	2.66	1.17	2.91	1.15	2.30	1.11
Beer	2.39	1.05	2.51	0.91	2.21	1.20
Soft drinks	2.99	1.09	2.88	1.01	3.15	1.19
Spirits	2.03	0.98	1.95	0.89	2.15	1.07
Ready to eat	2.44	1.04	2.20	0.91	2.79	1.13
Frozen food	2.76	0.79	2.72	0.64	2.82	0.96
Canned food	2.57	0.80	2.56	0.68	2.59	0.95

Means are calculated on the food frequencies of consumption ranging on a 5-point Likert scale (1 = Never; 2 = Less than once a week; 3 = A few times a week; 4 = Once a day; 5 = , More than once a day). *SD* standard deviation

Italy is characterized by a higher consumption frequency of grains, fruit and vegetables, fish, cold cuts, milk, wine and beer compared to the USA, where we instead find a higher average consumption frequency of eggs, salty snacks, and soft drinks. The consumption frequency of other foods is instead consistent in the two countries. Regarding the method of preservation and processing of foods, ready-to-eat foods are consumed more frequently in the USA.

As far as adherence to the Mediterranean diet is concerned, the MD index is clearly differentiated in the two countries and appears, on average, greater in Italy (MD index = 17.8) than in the USA (MD index = 13.8) (Table 3).

**Table 3:** Pre-COVID-19 MD Index in Italy and the USA

	Total sample		Italy		USA	
	Mean	SD	Mean	SD	Mean	SD
*MD Index*	16.17	4.38	17.81	3.92	13.80	3.90

*SD* standard deviation

The segmentation procedure started with the analysis of principal components. PCA made it possible to identify nine components (latent variables) whose variance was greater than or equal to 1. Table 4 shows the nine rotated components with loadings greater than 0.4.

**Table 4:** Components identified by means of PCA

Variable	Comp1	Comp2	Comp3	Comp4	Comp5	Comp6	Comp7	Comp8	Comp9
Grains									0.56
Legumes								0.87	
Potatoes							0.85		
Fish						0.83			
Red meat					0.45				
Pork meat					0.65				
Sweets				0.66					
Salted snacks				0.60					
Eggs	0.54								
Milk	0.60								
Dairy products and cheeses	0.43								
Wine			0.61						
Beer			0.55						
Soft drinks									− 0.55
Spirits			0.54						
Ready to eat		0.56							
Frozen food		0.55							
Canned food		0.54							

Table shows only factor loadings greater than 0.40

Eggs, milk, and cheese characterize and interpret the food behaviour of component 1; component 2 refers to the methods of food preservation (ready-to-eat, frozen, or canned); alcoholic beverages characterize component 3, while component 4 is made up of junk food; component 5 groups red meat and pork, and components 6, 7, and 8 specifically refer to the consumption of fish, potatoes, and legumes. The last component concerns grains and, inversely, is correlated with soft drinks.

Table 5 shows the average values in percentage variations in quantities of food consumed referring to post-lockdown compared with before the pandemic.

**Table 5:** Average values in percentage variations in quantities referring to post-lockdown compared with before the pandemic for the categories studied in the three clusters

Foods	Cluster 1 (Eaters) n = 493 (%)	Cluster 2 (Health-conscious) n = 322 (%)	Cluster 3 (Habituals) n = 413 (%)	Total sample (%)
Grains	72	25	2	38
Vegetables and fruit	94	68	6	60
Legumes	44	24	1	25
Potatoes	56	18	2	29
Fish	46	35	3	30
Processed meat	28	− 33	3	3
Red meat	38	− 16	2	12
Pork meat	25	− 19	2	5
Sweets	66	− 9	3	26
Salted snacks	55	− 18	1	18
White meat	63	26	4	35
Eggs	72	19	5	37
Milk	68	8	5	32
Dairy products and cheeses	72	13	7	35
Wine	42	− 35	3	8
Beer	36	− 42	7	5
Soft drinks	65	− 13	3	24
Spirits	22	− 61	− 7	− 11
Ready to eat	47	− 32	− 1	10
Frozen food	63	− 1	4	27
Canned food	51	− 12	2	18

The first group (Cluster 1), which is the largest (40%), is characterized by increased consumption in the quantity of all the foods considered. For grains, fruit and vegetables, eggs, and cheese, this increase is considerable (more than 70% compared to before the advent of COVID-19), while potatoes, sweets, salted snacks, white meats, milk, soft drinks, frozen and canned foods surpass the 50% mark. The subjects who make up this cluster have changed their dietary habits compared to their pre-COVID-19 behaviour. On the other hand, there was a clear worsening of the diet, as far as health and environmental sustainability is concerned. Numerous authors have stressed that an increase in the quantity of food consumed is generally harmful to health, having an impact on the body mass index (Czepczor-Bernat and Brytek-Matera 2021; Grant et al. 2021). It is also clear that the generalized increase in food consumption is burdensome in terms of sustainability (Steenson and Buttriss 2021). Based on the large increases in food consumption, this cluster has been labelled “Eaters”.

Cluster 2 (26%) was characterized by a marked increase in the consumption of the quantity of fruit and vegetables (+ 68%), a moderate increase (less than 30%) in the consumption of grains, legumes, potatoes, white meats, eggs, and cheese, and a decline in cold cuts, red meats, pork, salted snacks, wine, beer, soft drinks, hard liquor, ready-to-eat foods, and canned foods. Similar to “Eaters”, this cluster recorded structural variations in food consumption with respect to the advent of COVID-19, but unlike what was recorded for “Eaters”, these changes led to a clear improvement in the diet, be it in terms of health or in terms of environmental sustainability. In particular, the diet not only veered toward a more markedly plant-based model, in which the rapport between the quantity of vegetables and meat was greater, but there was also a marked reduction in the consumption of alcohol. This group of consumers reacted to the lockdown, assuming more virtuous and responsible behaviour, and their lifestyle approached a healthy and sustainable dietary model. For this reason, we have called this cluster “Health-conscious”.

For Cluster 3 (34%), the H1 hypothesis must instead be rejected. In fact, post-lockdown, food consumption returned to the pre-COVID-19 level for all the food types considered. The variations recorded are indeed minimal and can be considered nil (between − 10% and + 10%). We have therefore called this group of consumers “Habituals”.

The results of the cluster analysis make it possible to test Hypotheses H1 (The lockdown has induced changes in dietary habits that have been maintained even after the end of restrictions both in Italy and in the USA) and H2 (The lockdown has caused homogeneous changes in the dietary behaviours related to health and sustainability). The H1 hypothesis is valid for two out of three groups that have shown structural changes in food consumption. In this case, at the end of the lockdown, food consumption did not return to the same pre-COVID-19 level, suggesting new dietary habits for 66% of consumers. Moreover, the results obtained make it possible to also accept Hypothesis H2, showing that the changes observed have led to different dietary models with respect to the dietary characteristics in terms of healthiness and environmental sustainability. In particular, among those who changed their dietary habits, we clearly note two antithetical behaviours with respect to the diet’s overall healthiness and sustainability: those who after lockdown drew away from a healthy and sustainable diet and, conversely, those who assumed even more responsible behaviours, drawing even closer to a healthier diet in which the ratio between the consumption of vegetables and meat is greater.

This first series of results allowed us to identify those who changed or did not change their dietary behaviour after the end of the lockdown and enabled us to evaluate whether these changes brought them closer to or further away from a healthy and sustainable dietary model. It now seems particularly important to strengthen the analysis by checking the heterogeneity of results across demographic, psychographic, and behavioural characteristics.

The following tables are the result of CHAID analysis and represent the absolute and percentage distribution, per line in the three clusters of the independent variable. To understand the significance of the results of the following tables, it is useful to compare the datum of the distribution of the independent variable in the three clusters with respect to their overall representativeness.

The first variable tested was the one pertaining to the observance of the MD measured in the period prior to COVID-19. Using this information has permitted us to test the H3 hypothesis: the variations in dietary behaviours after lockdown depend on the previous diet. Table 6 shows the result of the CHAID with respect to the MD Index, which proves statistically significant for the three clusters. In this case, the greater the score of the MD Index, the better the observance of the Mediterranean style of diet.

**Table 6:** Classes of observance of the Mediterranean diet (MD Index) in the three clusters before the pandemic

MD Index	Eaters (%)	Health-conscious (%)	Habituals (%)	Total sample
1–13	53	23	24	335
14–18	44	28	28	497
19–20	32	29	39	194
21–28	24	40	36	203
Total	40	26	34	1229

LR chi-square = 34.95 df = 2 prob < 0.0001

The result in Table 6 points out how the low scores of the MD variable are the ones that characterize the “Eaters” (53% of the cases with an MD 1–13 score are present in this cluster against a numerousness of 40% with respect to total consumers). Remembering that “Eaters” were characterized by a marked increase in consumption, we can affirm that having a dietary style far from the Mediterranean diet has contributed to the assumption of less sustainable dietary models. In contrast, “Health-conscious” were characterized by a greater observance of the Mediterranean diet. Considering that this is the cluster that showed improvements in the diet after lockdown, it is confirmed that the previous dietary habits influenced the behaviours assumed with the return of the normal situation. “Habituals”, the cluster that did not change its diet in health terms, shows middle-high values of observance of the Mediterranean diet.

We therefore fail to reject the H3 hypothesis. In particular, those who followed a dietary style that did not conform to the Mediterranean diet structurally changed their food consumption after the lockdown, increasing the distance between themselves and a model of a sustainable diet. In contrast, the virtuous behaviours with respect to the healthiness of the dietary style observed in “Health-conscious”, after lockdown, are connected with a dietary behaviour that observes the Mediterranean diet that was already followed before COVID-19.

We continue here by profiling the clusters and by analysing the sociodemographic variables. In this context, the hypothesis to test is H4: The variations in dietary behaviours after lockdown depend on sociodemographic characteristics. The first significantly different variable among the three clusters was age (Table 7).

**Table 7:** Sociodemographic percentage differences of the clusters: age

Age	Eaters (%)	Health-conscious (%)	Habituals (%)	Total sample
< 52	54	21	25	725
52–71	26	38	36	420
> 71	11	50	39	84
Total	40	26	34	1229

LR chi-square = 126.96 df = 4 prob < 0.0001

In this case, the first group is clearly marked by younger subjects (54% of subjects under 52 are found precisely in this group), while “Health-conscious”, on the contrary, are clearly characterized by the presence of elderly people (50% of subjects over 71 against representativeness of this cluster of 26%). “Habituals” were also characterized by subjects over 52 years of age. From the viewpoint of age, the pandemic shock proved to have oriented most of the older population toward healthier and more sustainable dietary habits.

Significant differences between the clusters (Table 8) also occur from the viewpoint of country of origin.

**Table 8:** Sociodemographic percentage differences of the clusters: country of origin

Country	Eaters (%)	Health-conscious (%)	Habituals (%)	Total sample
IT	33	31	36	726
USA	54	25	21	503
Total	40	26	34	1229

LR chi-square = 55.89 df = 2 prob < 0.0001

“Eaters” are mainly represented by subjects who live in the USA, while “Health-conscious” and “Habituals” are mainly Italian. This result shows how the reaction to the shock of the lockdown principally and negatively impacted the American population, posing the theme of the motivations that brought on this result.

This result shows that, although departing from a different initial situation with respect to pre-pandemic habits in the two countries (Table 1), the shock of the lockdown brought about three models of change in food consumption, which presented in both countries, although with a different intensity. In particular, the American sample (54%) mostly falls in “Eaters”, demonstrating a worsening of the diet. Only 25% instead witnessed an improvement in the diet’s healthiness and sustainability. The remaining 21% returned to the same pre-pandemic consumption model. As far as the Italian sample is concerned, however, the most frequently assumed behaviour (36% of the sample) is that of a return to pre-pandemic behaviours. The remaining part of the sample is almost uniformly distributed among those who improved (31%) and worsened (33%) the healthiness and sustainability of their diet. The results thus point out that the shock of the lockdown in the USA corresponded to an overall worsening of the diet for a wide portion of people compared to what occurred in Italy.

Finally, Table 9 shows that following the lockdown, more virtuous behaviours were assumed among women than among men.

**Table 9:** Sociodemographic percentage differences of the clusters: sex

Sex	Eaters (%)	Health-conscious (%)	Habituals (%)	Total sample
Male	45	25	30	580
Female	38	32	30	649
Total	40	26	34	1229

LR chi-square = 7.19 df = 2 prob = 0.027

The data processing we have conducted thus far enables us to affirm that the variations in dietary behaviours after lockdown depend on the sociodemographic characteristics and therefore verify H4.

The last hypothesis to test, H5, concerns the possible impact of food choice motives on the variations in dietary behaviours after lockdown. Table 10 shows the food choice motives that proved significantly different in the three clusters.

**Table 10:** Percentage differences with respect to the FCMs in the three clusters

Variables and levels	Eaters (%)	Health-conscious (%)	Habituals (%)	Total sample
*Quick*				
1–3	34	34	32	507
4	44	25	31	547
5	56	23	21	175
(LR chi-square = 31.72 df = 4 prob < 0.0001)				
*Weight*				
1–3	34	29	37	469
4–5	45	29	26	760
(LR chi-square = 21.37 df = 2 prob < 0.0001)				
*Familiar*				
1–3	36	27	37	418
4–5	44	29	27	811
(LR chi-square = 15.80 df = 2 prob = 0.0015 (adj.))				
*State of mind*				
1–3	35	29	36	387
4–5	45	28	27	842
(LR chi-square = 13.15 df = 2 prob = 0.0056)				
*Healthy*				
1–3	44	21	35	
4–5	41	30	29	
(LR chi-square = 10.09 df = 2 prob = 0.025)				
*Fair*				
1–3	38	28	34	561
4–5	45	29	26	668
(LR chi-square = 10.09 df = 2 prob = 0.025)				
*Natural*				
1–3	58	18	24	60
4–5	41	29	30	1169
(LR chi-square = 7.55 df = 2 prob = 0.089)				
Total	40	26	34	1229

“Eaters” are particularly mindful of the speed of food preparation, the aspects of foods related to the comfort and personal gratification they give, the ethical characteristics, and one’s body weight. The salutary properties of the diet, in terms of healthiness or the naturalness of foods, instead appear relatively less important. For “Health-conscious”, the choice of foods is principally motivated by the healthiness and naturalness of foods and not by their preparation time. Finally, “Habituals” are not characterized by specific food motives.

Therefore, these results enable us to confirm H5, whereby FCMs have had an impact on the changes in dietary behaviours after lockdown.

The information obtained via the CHAID analysis provides the following insights, which delineate the principal impacts on dietary consumption connected with the end of the lockdown:

- Among American males under 50 years of age, we note a prevalence of subjects whose diet has worsened, both in terms of healthiness and in terms of environmental sustainability. These subjects adopted dietary choices that are quick to prepare, familiar, and gratifying
- A large number of subjects over 70, mostly Italians, have structurally improved their diets in terms of healthiness and sustainability.
- Most Italians between 50 and 70 years of age have maintained their dietary habits during the pre-COVID period.
- Subjects who before the pandemic followed the model of consumption furthest from that of the Mediterranean diet reacted to the shock by diminishing the sustainability of their food consumption.

## Discussions and policy implications

Our study has investigated how lockdowns have influenced the dietary habits of Italians and Americans, comparing pre-pandemic consumption with that of the period following the lockdown. The lockdown has represented an extensive and drastic event that imposed changes on people’s habits, at times even structurally, including, among others, as with other types of shock analysed in the literature, dietary habits.

The methodological approach that we utilized made it possible to test the five research hypotheses aimed at understanding how the unavoidable changes of these periods have affected people’s dietary habits and how they differentially impacted different groups of people. Specifically, three models were identified in response to the shock regarding food habits. These three models of consumption represent the three transversal adaptive behaviours that consumers assumed in the post-lockdown period, and they proved independent of socio-economic characteristics and pre-pandemic dietary habits. These characteristics instead considerably and significantly impact the probability of belonging to each adaptive model. In two cases, the diet changed with respect to before the pandemic, while in one case, consumption returned to the previous level. The successive profiling of groups was performed in view of delineating behavioural insights useful for policy-makers (Kuehnhanss 2019). The two groups that changed their dietary habits indeed present different characteristics, and one of them shows unsettling considerations concerning diet quality. Both groups are made up of Americans and Italians, a sign that the adaptive response to shock does not consider, in general terms, the sociocultural characteristics and the dietary habits before the pandemic, which were both profoundly different in the two countries. On the other hand, the composition concerning the two groups is quite different and points out a very different trend in the two countries’ modification of diet in a positive or negative sense with respect to healthiness and sustainability, such behaviour is also highlighted by Chenarides et al. (2020).

The shock of the lockdown induced a worsening of the diet both in terms of healthiness and in terms of diet sustainability in approximately 40% of the sample analysed. This group of consumers is mostly represented by subjects who live in the USA, who are males under the age of 50 with large family units. For this portion of the population, the effect is similar to what occurs during other shocks in which the subjects who live in developed countries find refuge in food to counter the distress tied to adverse events, generally increasing consumption (overeating) and, in particular, increasing the consumption of comfort food (emotional eating) (Adam and Epel 2007; Henderson et al. 2010; Kuijer and Boyce 2012; Ma et al. 2021). The diet thus acquires the role of a consolatory act, a refuge from external negativity. On the other hand, the negative effects on the economy and on employment during the lockdown reduced both economic resources and the availability of fresh foods, thereby diminishing the quality of diet and its adherence to the Mediterranean model. This effect is amplified precisely in a country such as the USA where even before COVID, the population showed a generally lesser familiarity with the Mediterranean diet, a food model universally considered to be among the healthiest. In this context, FCMs also play an important role, pointing out how for these subjects, the healthiness and naturalness of foods are still relatively unimportant purchasing criteria when compared to aspects tied to the convenience of food preparation, the level of comfort the foods give, and the degree of gratification they can produce. While this phenomenon characterized the consumption model of the USA by 54% of the sample living in the country, approximately one-third of the sample in Italy adopted this type of response to lockdown. This underlines the need to prepare information in advance aimed at public health education related to dietary choices during the lockdown or other disasters.

Approximately one-third of the sample increased the ratio of plant-based foods, thereby increasing the healthiness and sustainability of their dietary style. These are mostly Italians older than 70 who, already before COVID, adopted a diet close to the Mediterranean model. In this case, it emerges that the purchasing motivations of foods are tied to their healthiness and the naturalness and do not take into account quick preparation. The presence of this cluster of mainly elderly Italian people can be explained by the great culinary traditions of the country and by the fact that the pandemic had the most dramatic effects on this age bracket, thus probably also inducing greater attention to healthier lifestyles.

Shock did not affect the dietary habits of the remaining part of the subjects considered who indeed maintained the same levels of consumption as before the crisis. These are mostly Italians between the ages of 50 and 70 who did not pay any particular attention to the emotional aspect of food, food familiarity or concerns about body weight.

Identifying the different answers to the shock of the lockdown and profiling different groups makes it possible to better understand the behavioural dynamics and the impacts on dietary habits owing to this particular exceptional event; these answers provide useful and strategic information to aid in developing communication campaigns and food policies. The results of our work point out that people do not always act predictably, assuming rational decisions, but that their behaviour is determined by a series of often latent factors. In this sense, the field of behavioural science, which includes behavioural economy, psychology, and the other branches of social sciences, offers practical cues for the development of policies most in line with people’s decision-making processes. Political tools based on understanding people’s behaviours and choices and on the use of timely nudges tend to enable the development of strategies, policies, and measures that are more effective, accepted, appropriate, and fit to the purpose than traditional model-based policies (Aibana et al. 2017; Reisch 2021). The results that emerged from our analysis constitute a point of departure for a more extensive and coherent understanding of the variations in dietary habits stemming from the pandemic and can define the most adequate tools of food and health policy to orient dietary choices towards more sustainable and healthier habits. In this sense, a possible future development of this paper could precisely concern a broader definition of the behavioural insights (Grunert et al. 2021), involving other aspects related to values, lifestyles, and food attitudes. The main limitations of this paper instead concern the fact that the global pandemic had not yet ended by the time of our research and therefore could have still depended on the preoccupations stemming from it. In this sense, it might prove interesting to compare our results with the current situation to verify the permanence of the observed dynamics.

## Data Availability

Data and material will be available on request.

## Footnotes

1 Hierarchical methods are defined as those that, starting from a situation in which we have n clusters of a single unit, via the successive fusions of less distant clusters, we attain a situation in which we have a single cluster that contains all the n units. The final product of the hierarchical methods is therefore not a single partition of the n units, but a series of partitions that can be graphically represented by means of a dendrogram where the level of distance is reported on the axis of the ordinates, while the single units are reported on the axis of the abscissas. Each branch of the diagram (vertical line) corresponds to a cluster. The conjunction line (horizontal) of two or more branches identifies the level of distance at which the clusters fuse. The hierarchical methods are distinguished for the manner in which, after the p-nth fusion, the distances between the new cluster and the remaining ones are calculated. The hierarchical algorithms proposed in the literature (single link method, complete link method, median link method, centroid method, Ward method, etc.) are differentiated solely by the different criterion that regulates the assessment of the distances between the groups in view of the series aggregations (Johnson 1967; Everitt 1979).
